# Cholesterol Functionalized Nanoparticles Are Effective against *Helicobacter pylori*, the Gastric Bug: A Proof‐of‐Concept Study

**DOI:** 10.1002/adhm.202404065

**Published:** 2025-02-05

**Authors:** Ana Sofia Pinho, Renato Pereira, Mariana Pereira, Akhilesh Rai, Lino Ferreira, Maria Cristina Lopes Martins, Paula Parreira

**Affiliations:** ^1^ i3S ‐ Instituto de Investigação e Inovação em Saúde Universidade do Porto R. Alfredo Allen 208 Porto 4200‐135 Portugal; ^2^ INEB ‐ Instituto de Engenharia Biomédica Universidade do Porto R. Alfredo Allen 208 Porto 4200‐135 Portugal; ^3^ ICBAS ‐ Instituto de Ciências Biomédicas Abel Salazar Universidade do Porto Rua Jorge de Viterbo Ferreira 228 Porto 4050‐313 Portugal; ^4^ FEUP ‐ Faculdade de Engenharia Universidade do Porto Rua Dr. Roberto Frias Porto 4200‐465 Portugal; ^5^ CNC ‐ Centro de Neurociências e Biologia Celular Universidade de Coimbra Rua Larga 3004‐504 Portugal; ^6^ FMUC ‐ Faculdade de Medicina Universidade de Coimbra Azinhaga de Santa Comba (Celas) 3000‐548 Portugal; ^7^ CIBB‐ Centre for Innovative Biomedicine and Biotechnology Associate Laboratory Universidade de Coimbra Rua Larga 3004‐504 Portugal

**Keywords:** antibiotic‐free strategies, bioengineering, gold nanoparticles, *Helicobacter pylori*, lipids, self‐assembled monolayers

## Abstract

*Helicobacter pylori* chronic infection is the highest risk factor for the development of gastric cancer, being this Gram‐negative bacterium classified as carcinogenic. The mounting resistance of *H. pylori* to antibiotics calls for innovative therapeutic strategies. Here, the proof‐of‐concept studies that support the development of a “trojan horse” therapeutic strategy based on cholesterol‐grafted nanoparticles (Chol‐NP) to counteract *H. pylori* infection are depicted. The bacterium ability to specifically recognize and bind to surface grafted cholesterol is demonstrated by its adhesion to cholesterol(Chol)‐functionalized self‐assembled monolayers (SAMs) on gold substrates (2D Chol‐SAMs) in a concentration dependent manner, with optimal Chol‐SAMs prepared with 25% Chol‐polyethylene glycol (PEG)‐thiol in solution (75% tetra(ethylene glycol)‐thiol). These results further show that cholesterol functionalized gold nanoparticles (3D Chol‐SAMs, Chol‐NP) eradicate *H. pylori* at a minimum bactericidal concentration of 125 µg mL^−1^. Chol‐NP kill *H. pylori* through internalization and membrane rupture, as observed by transmission electron microscopy (TEM). Chol‐NP are cytocompatible (human gastric adenocarcinoma (AGS) cell line), non‐hemolytic and innocuous to bacteria representative of the gut microbiota (*Escherichia coli* and *Lactobacillus acidophilus*). This study supports the further development of cholesterol functionalized biomaterials as an advanced and targeted treatment for *H. pylori* infection.

## Graphical abstract

*Helicobacter pylori* gastric infection is widespread and recurrency is common, fuelled by bacterial resistance to antibiotics. Therefore, specific and noninducing resistance alternative treatments are an urgent need. Taking advantage of this gastric pathogen auxotrophy for cholesterol, a “trojan horse” strategy for *H. pylori* eradication is depicted by engineering cholesterol‐decorated nanoparticles.

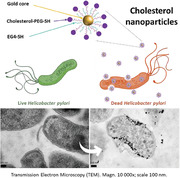

## 1 Introduction

Infection has emerged as a fundamental aspect in cancer, with a growing number of pathogens recognized as oncogenic and accounting for approximately 30% of cancer cases.^[^ ^1^ ^]^ The International Agency for Research on Cancer (IARC) classified 11 infectious pathogens as group 1 carcinogens (definite carcinogen).^[^ ^2^ ^]^ Among them is *Helicobacter pylori*, a Gram‐negative bacterium that infects the epithelial lining of the stomach of close to 4 billion people and, among other gastric disorders, is responsible for 90% of all gastric cancers, the 5^th^ most common and the 5^th^ deadliest worldwide.^[^ ^3^, ^4^, ^5^, ^6^ ^]^ The link between the effective and timely treatment of *H. pylori* and a severe reduction in the prevalence of gastric cancer is well established and, as such, eradication is an advocated preventive strategy.^[^ ^7^, ^8^, ^9^, ^10^ ^]^ The currently advised treatment is the bismuth quadruple therapy (BQT) [proton pump inhibitors (PPIs) or H_2_‐blocker + bismuth + two antibiotics] but the prolonged and excessive use of broad‐spectrum antibiotics combined with the patients lack of compliance to the complex therapeutic scheme, led to the emergence of *H. pylori* resistant strains and, consequently, therapeutic failure.^[^ ^1^, ^11^, ^12^, ^13^, ^14^ ^]^ Besides the mounting antibiotic resistance, the potential impact of widespread antibiotic use on gut microecology raises additional concerns.^[^ ^15^ ^]^ Accordingly, it has become increasingly risky to rely solely on conventional antibiotics and an urgent demand exists for new antibiotic‐independent strategies.

*H. pylori* has distinctive pathogenic features, namely its auxotrophic nature to cholesterol. As it is unable to biosynthesize this sterol *de novo*, to survive the bacterium must acquire it from the host gastric epithelial cells membranes. This cholesterol dependence promotes *H. pylori* positive chemotaxis and adherence to gastric epithelial cells, having specific receptors to recognize cholesterol (phosphatidylethanolamine) and a cholesterol‐α‐glucosyltransferase that enables cholesterol incorporation in its outer membrane as α‐glucosylated derivative.^[^ ^16^ ^]^ Cholesterol assimilation enhances the *H. pylori* membrane barrier, promotes evasion from the immune system and aids in the resistance against antimicrobial lipophilic compounds and antibiotics.^[^ ^16^, ^17^, ^18^ ^]^ In the gastric mucosa, cholesterol is found either bound to mucins or in the epithelial cell membrane, where it forms cholesterol‐rich microdomains, the lipid rafts, which serve as a platform for multiple cellular receptors and cell signaling cascades, including interferon‐γ, a key signaling molecule for summoning the adaptive immune system to the infected mucosa.^[^ ^19^ ^]^ By extracting cholesterol, *H. pylori* destroys these lipid rafts and generates a niche that supports bacterial survival shielded from the immune system, while inflammation continues unabated in the surrounding area.^[^ ^17^, ^18^, ^20^, ^21^, ^22^ ^]^ Besides, cholesterol extraction from gastric epithelial cells also accounts for their loss of integrity and further cellular damage, driving forces of tumorigenesis.^[^ ^22^, ^23^ ^]^

Previous work from Kobayashi et al. reported that cholestenone, a cholesterol metabolite, has been explored as an antibiotic against *H. pylori*, leading to the formation of a defective membrane. Cholestenone had antibacterial activity *i* *n vitro* against *H. pylori* ATCC^®^43504™ strain in a dose dependent‐manner and independently of the presence of cholesterol, as well as in vivo (C57BL/6 mice), where *H. pylori* SS1 infection was eradicated.^[^ ^24^ ^]^ However, in vitro assays show that *H. pylori* treated with cholestenone converted to a coccoid morphology, a stress‐response associated morphology.^[^ ^25^ ^]^ Therefore, although cholestenone appeared as promising, the induction of a stress morphology of this pathogen may not equal successful eradication and entails other risks, as the relapse of infection (within 1 year) post treatment, placing cholestenone under the same pitfalls experienced by the antibiotic‐based therapy.

Here, the proof‐of‐concept studies for the development of a “trojan horse” therapeutic strategy based on cholesterol‐grafted nanoparticles (Chol‐NP) against *H. pylori* are described. The rationale is that the grafted cholesterol will act as a bait for the bacterium, inducing the incorporation of the nanoparticle (NP) into its membrane, which will then compromise its integrity and lead to bacterium death. The bacterial ability to specifically recognize cholesterol‐functionalized surfaces was firstly studied using 2D model surfaces (self‐assembled monolayers—SAMs of alkanethiols on gold), as they are stable for biological assays, allow control at a molecular level and have been extensively used by us on the quest to develop novel therapies against *H. pylori*.^[^ ^26^, ^27^, ^28^, ^29^, ^30^, ^31^ ^]^ To evaluate the potential of this bioengineered strategy, namely its specific bactericidal performance against *H. pylori* and mechanism of action, as well as their cytocompatibility, cholesterol‐SAMs were prepared using 3D gold nanoparticles (Chol‐NP). Cholesterol‐SAMs (2D and 3D) were produced using different percentages of cholesterol polyethylene_75_‐thiol (Chol‐PEG) and tetraethylene glycol‐thiol (EG4; non‐fouling).

The advantage of using nanoparticles is its direct contact with the bacterial membrane, leading to structural changes, not being affected by mechanisms of genetic adaptation as the case of conventional antibiotics.^[^ ^32^ ^]^ In this case, using Chol‐NP as a decoy has the potential to target dormant cells (coccoid morphology, viable but non culturable *H. pylori*), once phosphatidylethanolamine (the receptor for cholesterol) is always expressed on *H. pylori* surface.^[^ ^33^ ^]^ This overcomes the need to have cells in exponential growth, as required for cholestenone and conventional antibiotics effectiveness. Also, it is expected that the interaction of *H. pylori* with Chol‐NP will minimize the use of the host cell cholesterol, which can aid in the recovery of the local immune response and reduce the massive use of drugs for gastric infection management.

In this proof‐of‐concept we described the *H. pylori* ability to recognize and adhere to surface grafted cholesterol moieties (2D) and demonstrated the bactericidal potential of cholesterol‐decorated nanoparticles (3D).

## 2 Results

### 2.1 Cholesterol 2D Surfaces (Chol‐SAMs) Characterization

**Figure 1:**
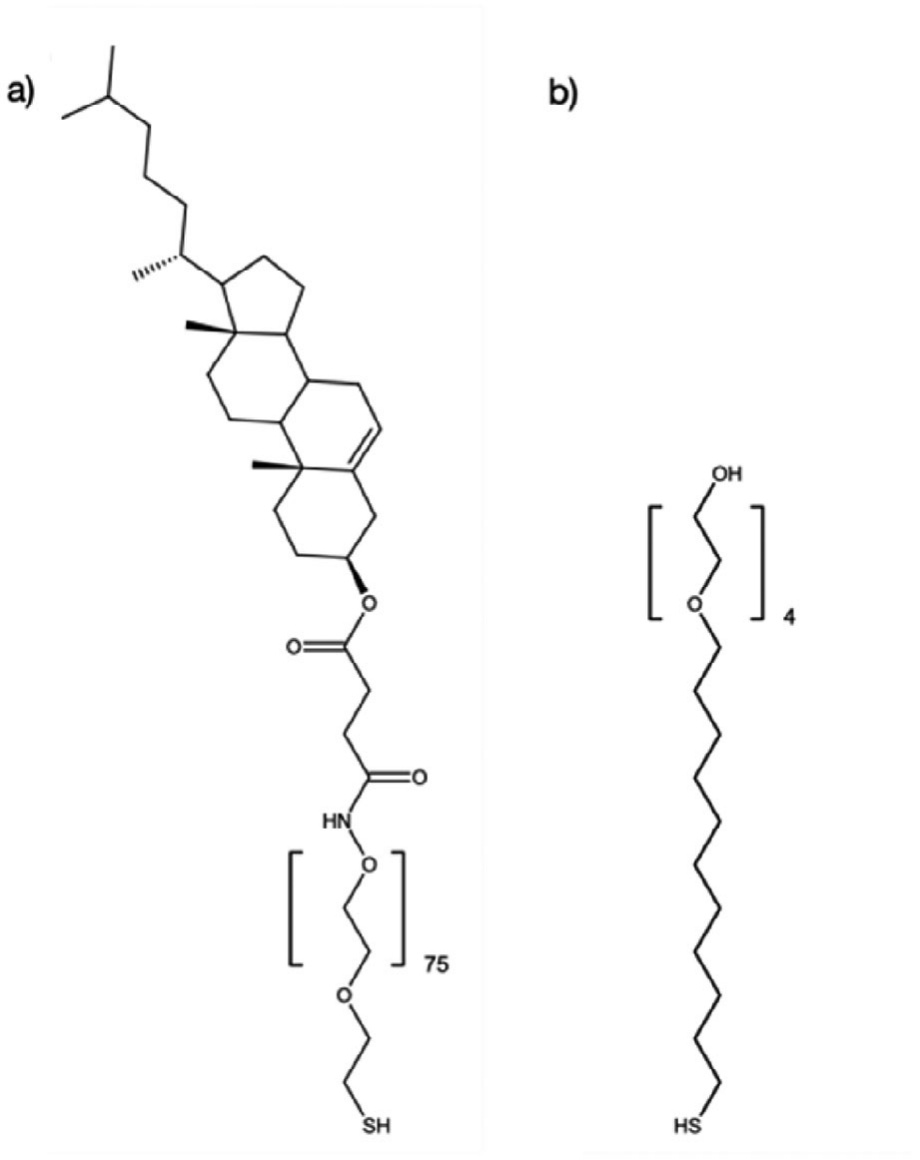
Molecular structure of (a) chol‐PEG (cholesterol‐poly(ethylene glycol)_75_ thiol/sulfhydryl) and (b) EG4 (1‐mercapto‐11‐undecyltetra(ethylene glycol) used in Chol‐SAMs.

**Figure 2:**
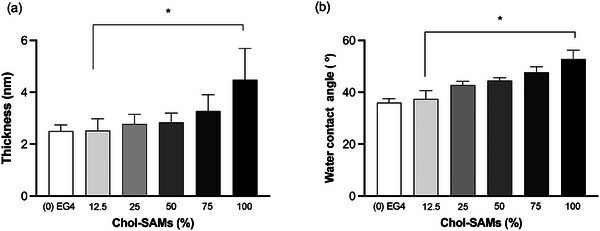
Chol‐SAMs characterization by (a) ellipsometry and (b) water contact angle. Chol‐SAMs (%) corresponds to the percentage of Chol–PEG in solution. Statistical analysis was performed using the one‐way ANOVA, followed by Tukey's multiple comparisons test. *n* = 3. *Statistically significant different from the control 0% Chol‐SAMs (EG4‐SAMs) (*p* < 0.05).

Cholesterol‐self‐assembled monolayers (Chol‐SAMs), with different % of cholesterol‐polyethyleneglycol_75_‐thiol (Chol‐PEG) and tetraethylene glycol‐thiol (EG4) (**Figure** **1**) on gold substrates, were successfully prepared as confirmed by ellipsometry, water contact angle (**Figure** **2**) and X‐ray Photoelectron Spectroscopy (XPS) analysis (**Table** **1**).

**Table 1:** XPS characterization. Relative percentage of chemical elements for Chol‐SAMs, calculated from their high‐resolution spectra. Chol‐SAMs (%) corresponds to the percentage of Chol‐PEG in solution.

Chol‐SAMs [%]	N1s	C1s	O1s	S2p
0	0.00	73.8	24.6	1.6
12.5	0.02	72.2	25.9	1.9
25	0.47	71.2	26.2	2.2
50	0.59	70.8	26.7	1.9
100	0.83	69.5	28.5	1.2

The average thickness of the model surfaces increased as the % of Chol‐PEG increased from 2.5 ± 0.1 nm (0% Chol‐SAMs) to a maximum thickness of 4.5 ± 1.2 nm (100% Chol‐SAMs) (Figure 2a), which is explained by the longer chain of the Chol‐PEG regarding to EG4, which was used to dilute cholesterol on the surface. This is indicative of the presence of Chol‐PEG in different ratios. The increase of the hydrophobicity, demonstrated by the increase of the water contact angle from 36° (0% Chol‐SAMs) to 53° (100% Chol‐SAMs, Figure 2b) also suggests the presence and exposure of cholesterol in different ratios. XPS surveys (data not shown) of 2D Chol‐SAMs showed only the anticipated elements (nitrogen (N), carbon (C), oxygen (O), sulfur (S), and gold (Au)) demonstrating no surface contamination. The relative elemental composition of each surface is given in Table 1. The presence of N1s can be used to detect the coverage of Chol‐PEG due to its presence on Chol‐PEG and its absence in the EG4 molecule (Figure 1). As expected, N1s was not detected on 0% Chol‐SAMs (EG4‐SAMs) but it increased with addition of Chol‐PEG in solution. The coverage of Chol‐PEG can be estimated in ≈57% for 25% Chol‐SAMs (≈71% for 50% Chol‐SAMs) when compared to 100% Chol‐SAMs. There is also a small decrease of the relative % of C1s and a small increase in O1s. The decrease in S2p from 2.15 (25% Chol‐SAMs) to 1.22 (100% Chol‐SAMs) is linked to the increase in the surface thickness and packing, which leads to signal attenuation of S.^[^ ^34^ ^]^ The characterization of the EG4‐SAMs (0% Chol‐SAMs) is in accordance with previous results.^[^ ^30^, ^35^ ^]^ Overall, surface characterization demonstrated that Chol‐SAMs with different cholesterol ratios were successfully obtained and that a minimum of 25% of Chol‐PEG in solution was necessary to have grafted cholesterol on the surface as observed by XPS data.

### 2.2 Cholesterol Nanoparticles Characterization (Chol‐NP, 3D Chol‐SAMs)

Cholesterol nanoparticles were synthesized by heating a solution of sodium citrate, potassium carbonate and tannic acid to which chloroauric acid was added, forming gold nanoparticles (Au‐NP).^[^ ^36^ ^]^ These were then functionalized with either EG4 and Chol‐PEG (Chol‐NP) or only EG4 (EG4‐NP). All nanoparticles were characterized in terms of size, polydispersity index, zeta potential, as well as their stability in acidic pH.

#### 2.2.1 Size, Polydispersity Index (PdI), and Zeta Potential

**Figure 3:**
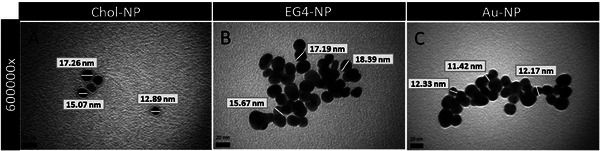
TEM images of Chol‐NP and NP controls (EG4‐NP and Au‐NP). NP diameter was measured in triplicate and is shown in the images. Magnification: 600000×. Scale bars: 20 nm.

Chol‐NP were characterized in water (Milli‐Q water) concerning size and PdI with dynamic light scattering (DLS), and zeta potential using electrophoretic light scattering (ELS). Results are given in **Table** **2**. NP morphology was analyzed with Transmission Electron Microscopy (TEM) (**Figure** **3**).

**Table 2:** NP characterization (size, PdI and zeta potential). Size and PdI were determined using dynamic light scattering (DLS) and zeta potential using electrophoretic light scattering (ELS). Data are expressed as mean ± standard deviation (SD) (*n* = 3, in triplicate).

NP	Size [nm]	PdI	Zeta potential [mV]
Chol‐NP	26.2 ± 0.6	0.4 ± 0.1	−1.5 ± 0.6
EG4‐NP	28.4 ± 0.6	0.4 ± 0.1	−15 ± 1.2
Au‐NP	9.1 ± 0.5	0.4 ± 0.3	−30 ± 0.8

Chol‐NP and EG4‐NP average size was higher than their original Au‐NP core (Table 2) and, together with the increase in zeta potential, confirm the successful functionalization of the Au‐NP with Chol‐PEG/EG4. While Au‐NP are surrounded by negative ions at the slipping plane, leading to a negative zeta potential (−30 mV), the presence of an EG4 chain (−15 mV) and Chol‐PEG increase the Chol‐NP zeta potential to a neutral value (−1.5 mV) as expected.^[^ ^37^, ^38^ ^]^ This difference in the zeta potential between EG4‐NP and Chol‐NP also reinforces the success of Chol functionalization. Despite their neutral value, all the NP had similar PdI (0.4), being considered polydisperse in accordance with the ISO 22412:2017 (PdI above 0.07) and as stated by Malvern Panalytical (PdI above 0.1).^[^ ^39^ ^]^ The absence of aggregation in water could be linked to the long ethylene glycol chain (*n* = 75) of the Chol‐PEG that may act as a surfactant, and as such, as a stabilizing coating, helping to maintain the Chol‐NP disperse.^[^ ^40^ ^]^ TEM images (Figure 3) show that all NP had spherical shape and similar size. NP had a clear contrast against the background, with Chol‐NP having a ring around the dark surface of the gold core, probably due to the presence of the hydrophobic sterol (Figure 3A). The slightly lower size of Chol‐NP and EG4‐NP reported by TEM compared with DLS are due to intrinsic differences associated to the techniques: while TEM measurements are obtained directly from the images (dry conditions), in DLS the NP size is determined by a hydrodynamic radius based on the diffusion of the nanoparticles in solution that may lead to a slight size overestimation.^[^ ^41^ ^]^ Although TEM images also suggest NP aggregation, this is attributed to the drying process. NP aggregation was not observed in DLS characterization or in TEM when the samples were previously embedded in resin and sectioned into slices (Figure 8).

#### 2.2.2 pH Stability in Gastric Settings

**Figure 4:**
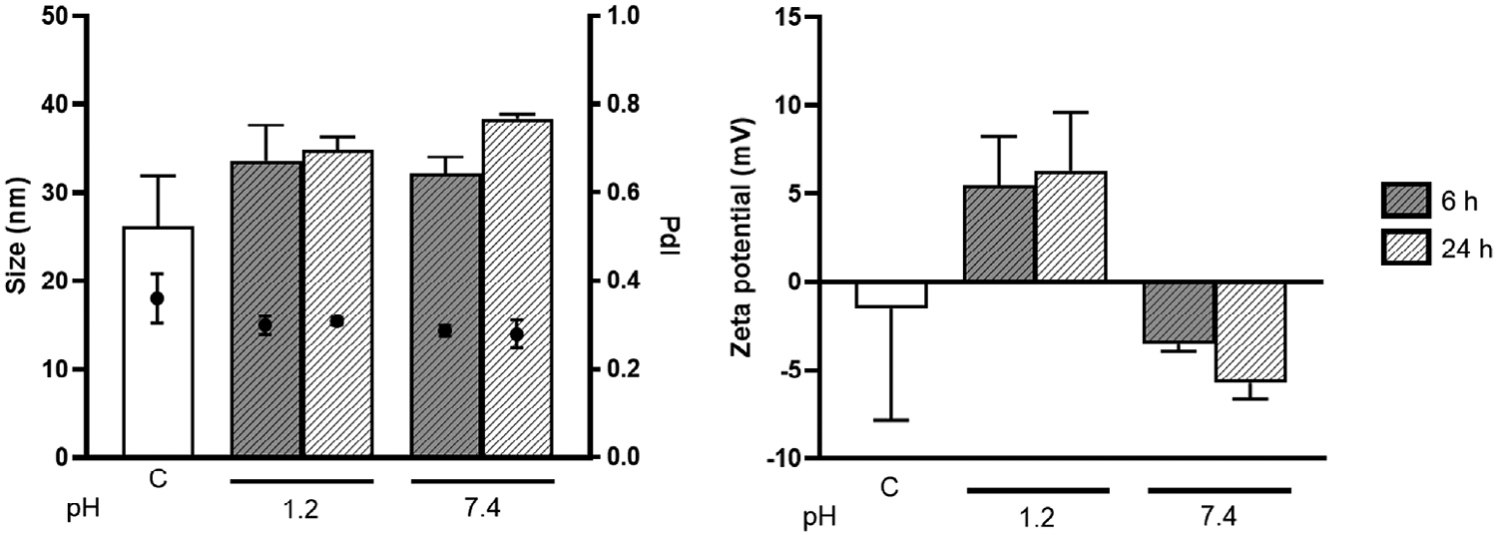
Chol‐NP characterization at different pH for 6 and 24 h. pH 1.2 (simulated gastric fluid, SGF) and 7.4 (phosphate buffer saline, PBS). C‐control measured prior to incubation at different pH. Values are mean ± SD. Statistical analysis was performed using the unpaired T‐test (*p* < 0.05). *n* = 3.

Chol‐NP stability was assessed by changes in size, PdI and zeta potential after their immersion in simulated gastric media mimicking the pH range in its opposite extremes (7.4 and 1.2) found within the stomach and over 2 time points (6 and 24 h) (Figure 4). The control NP (EG4‐NP) were also characterized (Figure S1, Supporting Information).

No significant differences in size and charge were observed and, despite the slight increase on the zeta potential at pH 1.2 (as expected), values remained close to neutral |10mV|.^[^ ^42^ ^]^ The PdI also remained below 0.4 in all conditions tested, indicating a monodisperse suspension, probably linked to the surfactant effect of the long ethylene glycol chain (*n* = 75) of the Chol‐PEG.^[^ ^40^, ^43^ ^]^ Altogether, these results indicate that Chol‐NP are stable in gastric‐like pH.

### 2.3 *H. pylori* Recognition of Immobilized Cholesterol (2D Chol‐SAMs)

#### 2.3.1 *H. pylori* Adhesion

**Figure 5:**
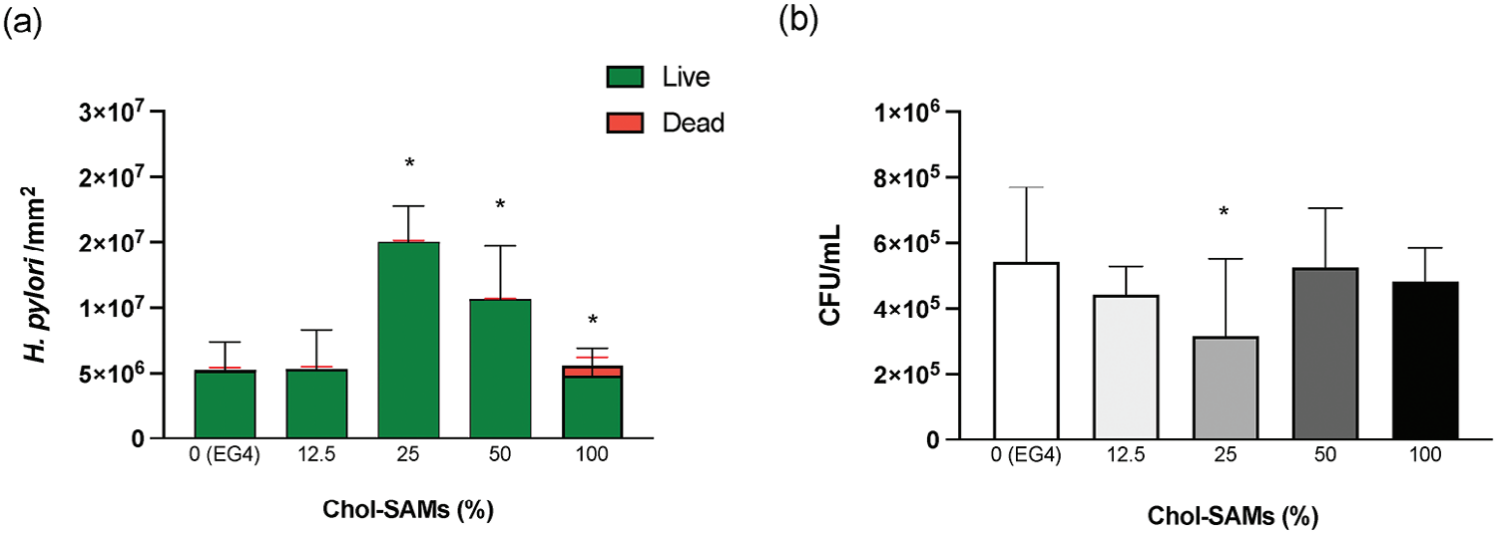
*H. pylori* J99 after 2 h of incubation with 2D Chol‐SAMs in PBS: (a) surface‐adherent bacteria, quantified after staining with LIVE/DEAD BacLight kit; total adhesion corresponds to the number of live (green) + dead (red) bacteria and b) planktonic/non‐adherent bacteria quantified by supernatant plating and CFU counting. Values are mean ± SD. Statistical analysis was performed using the One‐way ANOVA, followed by Tukey's multiple comparisons test*. n* = 3. *Statistically significant different from the control surface 0% Chol‐SAMs (EG4‐SAMs) (*p* < 0.05).

**Figure** **5** shows the adhesion of *H. pylori* J99 strain to 2D Chol‐SAMs (Figure 5a) as well as planktonic bacteria (Figure 5b) after 2 h incubation.

*H. pylori* adhesion was higher on 25% Chol‐SAMs compared to the other surfaces (Figure 5a). Conversely, less bacteria were found on the supernatants corresponding to the 25% Chol‐SAMs (Figure 5b), in agreement with the surface‐adhesion results (Figure 5a). Fewer bacteria adhered to the 100% Chol‐SAMs than to 0% Chol‐SAMs (EG4‐SAMs), suggesting that a surface highly packed with cholesterol may not render specific bacterial recognition. Although EG4‐functionalized surfaces are described as non‐fouling surfaces, i.e., resistant to cell and protein adhesion, *H. pylori* adhered to EG4‐SAMs, which is in accordance with what was previously described by us.^[^ ^29^, ^44^ ^]^ Also, bacteria viability was not compromised after adhesion (mortality did not exceed 20%) (Figure 5a).

##### Effect of Cholesterol in Solution

**Figure 6:**
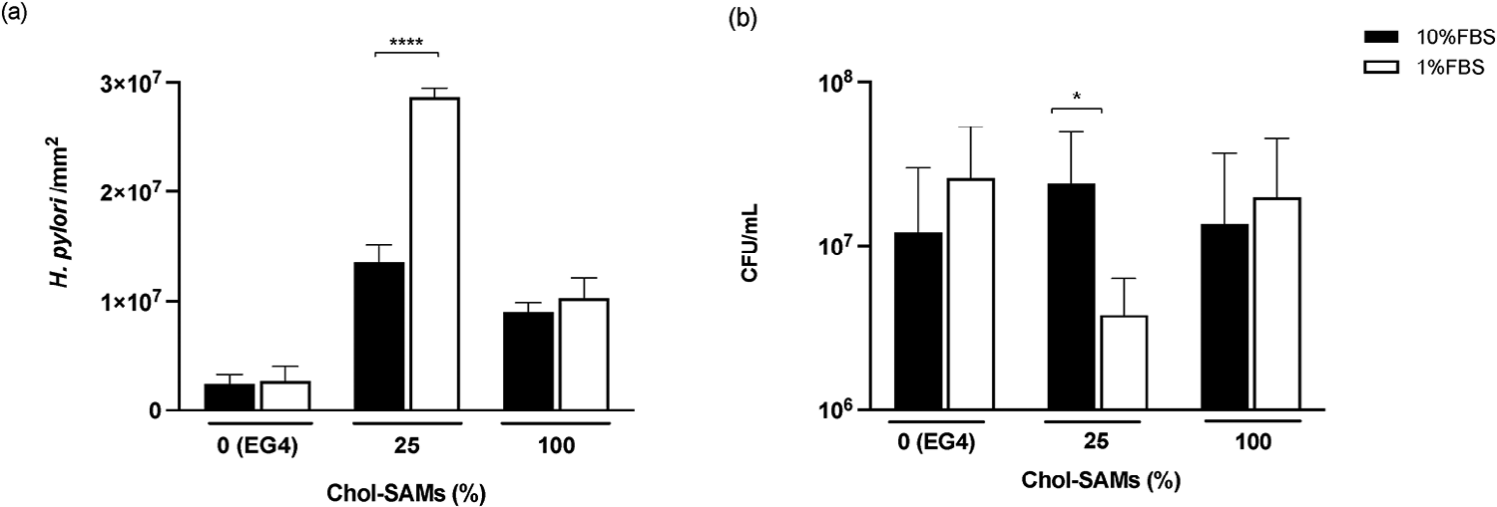
Effect of cholesterol (10% FBS vs 1% FBS) onto *H. pylori* J99 adhesion to 2D Chol‐SAMs after 2 h. (a) Adherent bacteria; (b) planktonic bacteria. Values are mean ± SD. Statistical analysis was performed using the unpaired T‐test with Welch's correction. *n* = 3. *Statistically significant different (*p* < 0.05). ****Statistically significant different (*p* < 0.0001).

The effect of competitive cholesterol in solution on *H. pylori* adhesion to 2D Chol‐SAMs was assessed by performing adhesion assays in the presence of 10% fetal bovine serum (FBS, source of cholesterol) versus 1% FBS (**Figure** **6**).

The bacterial adhesion was always higher to the 25% Chol‐SAMs than to 0 and 100% Chol‐SAMs, independently of the FBS concentration (Figure 6a). However, when less cholesterol was available (1% FBS), *H. pylori* adhesion to the 25% Chol‐SAMs increased compared to the same surface in 10% FBS (Figure 6a) accompanied by a pronounced decrease on the number of supernatant bacteria (Figure 6b). Contrarily, changes on the concentration of FBS did not alter the adhesion pattern for the 0% Chol‐ or 100% Chol‐SAMs (Figure 6a,b), showcasing the *H. pylori* specific adhesion for surface grafted cholesterol in a concentration‐dependent way.

##### Specific Adhesion

Chol‐SAMs selectivity for *H. pylori* (specific adhesion) was evaluated by using two bacteria representative of normal gut microbiota, *Escherichia coli* (Gram‐negative) and *Lactobacillus acidophilu*s (Gram‐positive).^[^ ^45^, ^46^ ^]^ Results (Figure S2, Supporting Information) demonstrated that both *E. coli* and *L. acidophilus* did not show affinity to cholesterol but only adhered following the increase of hydrophobicity (higher percentages of cholesterol) without significant differences between them and in accordance to what is reported in the literature.^[^ ^47^, ^48^ ^]^

### 2.4 Antibacterial Performance of Chol‐NP (3D Chol‐SAMs)

**Figure 7:**
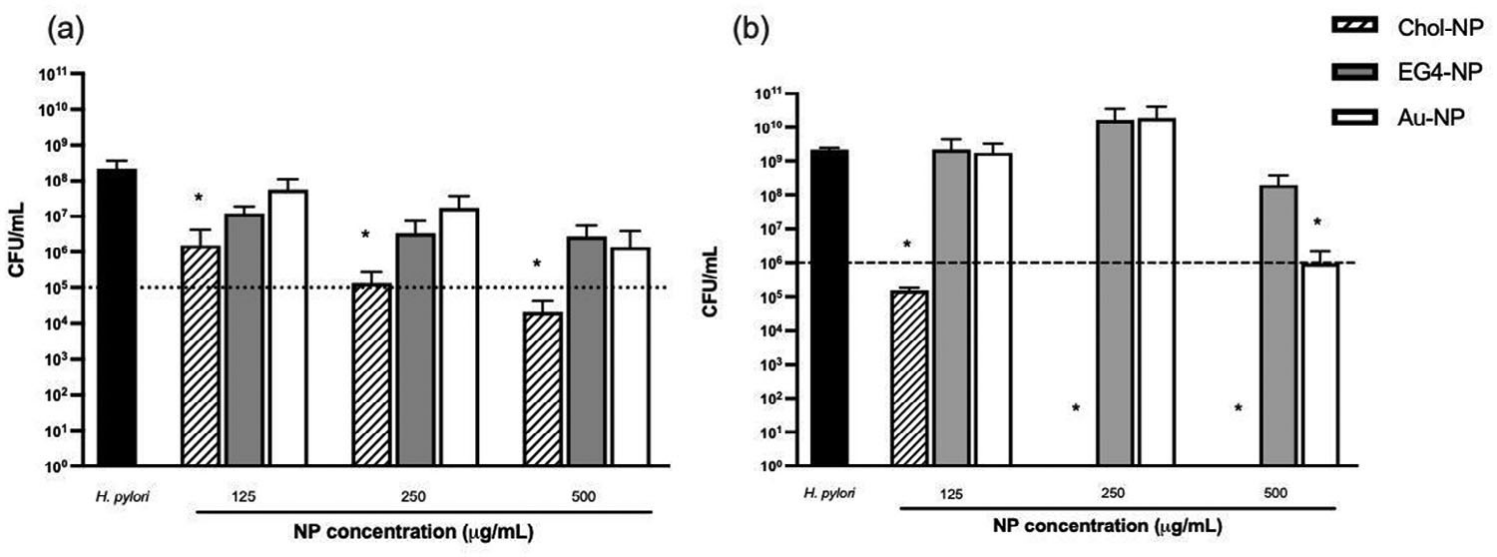
*H. pylori* J99 growth after incubation with Chol‐NP, EG4‐NP and Au‐NP over (a) 6 h and (b) 24 h. Assays were performed in BB+10% FBS. Values are mean ± SD. Statistical analysis was performed using the one‐way ANOVA, followed by Dunnett's multiple comparisons test*. n* = 3. *Statistically significant differences from the control (*H. pylori*), (*p* < 0.05). Dotted line indicates the bactericidal threshold (3 log reduction).^[^ ^49^ ^]^

The effect of Chol‐NP on *H. pylori* J99 growth was evaluated using different concentrations of nanoparticles (from 125 to 500 µg mL^−1^) after 6 and 24 h. EG4‐NP and Au‐NP were used as control. Results are shown in **Figure** **7**.

After 6 h (Figure 7a), Chol‐NP were bactericidal at 500 µg mL^−1^. This bactericidal effect was seen at all the concentrations tested after 24 h (Figure 7b). Also, free Chol‐PEG (at 1 mg mL^−1^, the concentration used to synthesize the NP) and incubated under the same conditions, did not affect *H. pylori* growth (data not shown) demonstrating the importance of the cholesterol exposure from the NP to achieve the bactericidal effect. Concerning the controls (Au‐NP and EG4‐NP), only Au‐NP were bactericidal after 24 h at the maximum concentration tested (500 µg mL^−1^), while no antibacterial effect was seen for EG4‐NP at the concentrations tested (Figure 7).

**Figure 8:**
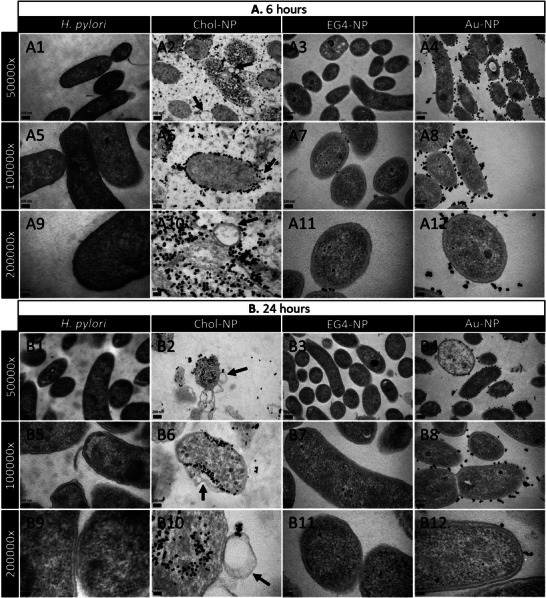
TEM images of *H. pylori* after exposure to Chol‐NP, EG4‐NP and Au‐NP at 125 µg mL^−1^ during A) 6 h and B) 24 h. Magnifications: 50.000x (A/B 1–4), 100–000x (A/B 5–8) and 200.000x (A/B 9–12). Scale bars: 200 nm (A/B 1–4), 100 nm (A/B 5–8) and 50 nm (A/B 9–12). Black arrows point towards deformations of the *H. pylori* bacterial membrane.

To investigate the mechanism of action of Chol‐NP, the interaction of NP at the minimum bactericidal concentration (MBC) of Chol‐NP (125 µg mL^−1^) with *H. pylori* membrane was studied using TEM (**Figure** **8**).

*H. pylori* exposed to Chol‐NP (125 µg mL^−1^) for 6 and 24 h (Figure 8A,B, respectively) display a high number of Chol‐NP around and within the bacteria. Moreover, bacteria exposed to Chol‐NP show cell membrane irregularities, such as membrane disruption and vesicle formation (black arrows Figure 8 (A2, A6, A10) and Figure 8 (B2, B6, B10)), which correlates with the decrease in CFU mL^−1^ observed in the previous assay (CFU mL^−1^ at Figure 7a,b). Although several Au‐NP were observed surrounding bacteria membrane, there is no internalization or visible effects onto the bacteria morphology (Figure 8 (A4, A8, A12) and Figure 8 (B4, B8, B12). Concerning EG4‐NP, very few NP were observed in the bacteria vicinity as well as no alterations on the bacteria membrane, probably because effects were only seen at higher concentrations (500 µg mL^−1^) (Figure 8 (A3, A7, A11) and Figure 8 (B3, B7, B11)).

Although the 3 types of NP were incubated at the same concentration (125 µg mL^−1^), more nanoparticles can be observed in the TEM images of Chol‐NP (Figure 8 (A2, A6, A10, B2, B6, B10)). This is likely due to the higher affinity of *H. pylori* towards these nanoparticles, translated in more Chol‐NP interacting with the bacterium membrane, being some even internalized. On the other hand, *H. pylori* was not attracted towards EG4‐NP and Au‐NP. Therefore, Chol‐NP were less likely to be removed after the centrifugation and supernatant discard step in the sample preparation protocol, unlike EG4‐NP and Au‐NP that were likely discarded, resulting in less NP on the TEM images. At *H. pylori* bactericidal concentration, Chol‐NP had no effects against *E. coli* and *L. acidophilus*, two representative bacteria of the gut microbiome (Figure S3, Supporting Information). These results hint that Chol‐NP are selective for *H. pylori*, suggesting their safety for gut microbiota.

### 2.5 Cyto‐ and Hemocompatibility of Chol‐NP (3D‐Chol‐SAMs)

**Figure 9:**
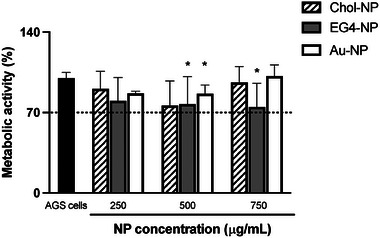
Metabolic activity of AGS cells after exposure to NP. Metabolic activity is expressed as the percentage of the cell metabolic activity of treated cells in relation to cells in culture medium only. Negative control: AGS cells; positive control (not shown): AGS cells incubated with 10% (v/v) solution of 30%V H_2_O_2_. Full RPMI medium was used as blanks for fluorescence emission subtraction. Values are mean ± SD. Statistical analysis was performed using the One‐way ANOVA, followed by Tukey's multiple comparisons test*. n* = 3. *Statistically significant different from positive control (*p* < 0.05).

Chol‐NP cytotoxicity was evaluated against a human gastric adenocarcinoma (AGS) cell line (**Figure** **9**) and according to the ISO 10993‐5:2009 standard.^[^ ^50^ ^]^

The general non‐cytotoxicity profile of Chol‐NP at the MBC (Figure 9) was confirmed, with cell viability values above the 70% threshold in accordance with the ISO standards.^[^ ^51^ ^]^ Moreover, AGS cells exposed to Chol‐NP retained their “healthy” epithelial morphology with a polygonal shape (mushroom‐like) that, in healthy conditions, demonstrated elongation and the formation of a monolayer. The negative control (only cells) had similar morphologies and confluency, highlighting that the Chol‐NP did not negatively affect the cells, reinforcing the cytocompatible profile of Chol‐NP even at 6X the bactericidal concentration.

The non‐hemolytic profile of Chol‐NP at the MBC was determined using a Hemolysis Assay on Human Red Blood Cells (RBC). Results are shown in **Table** **3** and the values for in vitro hemolysis are below the 5% threshold in accordance with the ISO standards.^[^ ^51^ ^]^ This is an important feature since *H. pylori* infected individuals will develop active peptic ulcers in which bleeding is a common complication.^[^ ^52^ ^]^

**Table 3:** Hemolysis percentage (%) determined by hemoglobin release after incubation with Chol‐NP (125, 250 µg mL^−1^), EG4‐ and Au‐NP (125, 250, and 500 µg mL^−1^). Controls with PBS (lysis negative control) and Triton 1% in PBS (positive control for 100% hemolysis), as well as controls with only NP in PBS were performed for each concentration tested (data not shown). Data are expressed as mean ± SD (*n* = 3, in triplicate).

Concentration [µg mL^−1^]	Chol‐NP [%]	EG4‐NP [%]	Au‐NP [%]
125	0 ± 0	2 ± 2	0 ± 0
250	1 ± 1	29 ± 10	0 ± 0
500	–	61 ± 5	14 ± 9

As expected, cells exposed to H_2_O_2_ showed extensive cell lysis and vacuolization, with less than 1% of the cells metabolically active (positive control, not shown).

## 3 Discussion

The antibiotic resistance patterns and antibiotics irreversible effect on the intestinal microecology of patients have led to the exploration of alternatives to counteract *Helicobacter pylori* chronic infection.^[^ ^53^, ^54^, ^55^, ^56^ ^]^

The ability to extract cholesterol from the host mucins and gastric cells plays a critical role in the development of *H. pylori* antibiotic resistance, bacteria resilience and the establishment of chronic infection that may persist for decades despite a vigorous host immune response, since this extraction process disrupts the lipid rafts, key elements of adaptative immune response.^[^ ^23^ ^]^ Although the cholesterol dependence is a feature found on other human pathogenic bacteria, as *Mycobacterium tuberculosis* and, even though other gut bacteria can link cholesterol,^[^ ^57^ ^]^ they are not auxotrophic to it, which makes a cholesterol‐based strategy targeted against *H. pylori* appealing on the route for new therapeutic options. While some strategies have resourced to cholesterol metabolites, as cholestenone, to induce the formation of a defective membrane and kill the bacteria, some of the core issues that plague antibiotics effectiveness still lingered, as the induction of coccoid‐shaped *H. pylori*, a more robust form used as a survival strategy.^[^ ^24^, ^25^ ^]^

Here the potential of cholesterol‐grafted nanoparticles (Chol‐NP) as a new strategy to counteract *H. pylori* based on a “trojan horse” approach was depicted, where the grafted cholesterol attracted the bacteria followed by the incorporation of the nanoparticles (NP), membrane disruption and bacteria death. Oral delivery strategies for gastric settings benefit from the use of a bioengineered approach as NP, as it allows to overcome gastric bioavailability and effectively reach *H. pylori* infection site (under the gastric mucus layer).^[^ ^58^ ^]^ Another interesting aspect of bringing NP into the equation is that most of the antibiotics resistance mechanisms are irrelevant for NP, as their mode of action is direct contact with the bacterial cell wall, leading to structural changes without the need to penetrate the cell and not affected by mechanisms of genetic adaptation.^[^ ^32^ ^]^ While cholesterol is a key biomolecule in several biological processes and may even promote tumorigenesis, this drawback is circumvented by the covalent grafting of the molecule onto a biomaterial: not only this process hinders the enzymatic recognition site, it also prevents leaching of the molecule, which remains linked to the NP and unavailable to enter other cellular processes.^[^ ^59^ ^]^ This rationale, namely the capacity of *H. pylori* to specifically recognize and bind to surface grafted cholesterol was validated using 2D self‐assembled monolayers of alkanethiols on gold ‐SAMs (assembled onto planar gold surfaces), since they easily allow the control of cholesterol orientation, exposure and concentration from a surface. Their selective bactericidal capacity as well as their mechanism of action against *H. pylori* was validated using 3D‐Cholesterol SAMs (assembled on gold nanoparticles) that are easy to synthesize, have high surface to volume ratio and are biocompatible.^[^ ^26^, ^60^, ^61^, ^62^, ^63^, ^64^ ^]^

Studies using 2D models (2D Chol‐SAMs) were essential to confirm *H. pylori* recognition of grafted cholesterol. We established that *H. pylori* J99 strain (ATCC 700824) (highly pathogenic strain) recognized and bound to Chol‐SAMs in a concentration‐dependent manner, being the optimum adhesion obtained for SAMs prepared with 25% Chol. These SAMs have around 50% of cholesterol coverage, demonstrating that higher ligand concentrations cannot be recognized by the target, and thus, the importance of using EG4 (non‐fouling) to aid in the exposure and proper dispersal of the cholesterol group trough the surface, promoting recognition (adhesion). Although *H. pylori* has been shown to adhere to either highly hydrophilic and hydrophobic surfaces, the considerably lower adhesion of the bacterium to 100% Chol‐SAMs may result from the high cholesterol sorting on this surface, which may hinder the recognition of the cholesterol groups in the monolayer.^[^ ^35^ ^]^ Furthermore, *H. pylori* adhesion to 2D Chol‐SAMs increased with the decrease of soluble cholesterol in the medium (from 10% down to 1% FBS, cholesterol source), demonstrating *H. pylori* recognition of the exposed cholesterol. Moreover, Chol‐SAMs selectivity to *H. pylori* was also highlighted by using other non‐cholesterol dependent bacteria, that adhered to this Chol‐functionalized surfaces based only on the increase on surface hydrophobicity. In opposite to *H. pylori*, these bacteria had higher adhesion to 100% Chol‐SAMs, surfaces where cholesterol moieties were not arranged to promote the specific recognition and binding.

The bactericidal potential of our strategy was then evaluated using 3D Chol‐SAMs (Chol‐NP). Monodisperse and round Chol‐NP were successfully prepared with sizes around 15 nm and Zeta potential of around −1.5 mV. These NP were stable in gastric‐like pH and, as such, are suitable for gastric applications. Chol‐NP killed *H. pylori* J99 through internalization & membrane disruption (TEM), with a minimum bactericidal concentration (MBC) of 125 µg mL^−1^ (at least 4 times lower than AuNP control ≥ 500 µg mL^−1^). Although this value is 4–8 times higher than the MBC reported for antibiotics/antimicrobial peptides, which may range from 16–32 µg mL^−1^, a direct comparison is not possible since cholesterol was bound onto AuNP.^[^ ^29^ ^]^ Also, most of the strategies reported in the literature that use AuNP are coupled to antibiotics, using these NP to enhance their performance.^[^ ^63^ ^]^ In addition, the size of NP largely influences the antimicrobial outcome. Gopinath et al. showed that the antimicrobial activity of AuNP treated with the dried fruit extract of *Tribulus terrestris* against *H. pylori* was largely dependent on the size of the NP, with smaller (<7 nm) particles achieving better MIC values than larger particles (55 nm), but sizes <10 nm raise the question of toxicity and bioaccumulation.^[^ ^63^, ^65^ ^]^ Testing cell cytotoxicity is a critical step in the proof‐of‐concept phase for designing new nanotherapeutics, as it provides valuable information on their safety and potential toxic effects. Moreover, *H. pylori* infection is linked to the development of peptic ulcers (15–20% of infected individuals) in which bleeding is the most common complication and new therapeutic approaches should also be evaluated regarding their hemolytic profile.^[^ ^52^ ^]^ The herein developed Chol‐NP, with around 15 nm, were cytocompatible towards human gastric adenocarcinoma (AGS) cell line, even at 6x the MBC (750 µg mL^−1^) and were not hemolytic against human red blood cells in at least 2× the MBC. While in vitro cytotoxicity tests cannot fully replicate in vivo conditions, they provide early warning signs of potential systemic toxicity or high likelihood of causing adverse effects in animal models or humans.

The different kinetics and effectiveness of Chol‐NP at lower concentrations compared to the controls (EG4‐NP and Au‐NP) suggests a higher interaction between the cholesterol groups and the bacteria, highlighting their targeting potential. Notably, as shown by TEM micrographs, the conversion to a coccoid morphology in the presence of Chol‐NP was not observed, which may allow overcoming the issue of the relapse of infection. Furthermore, and as expected by the 2D assays, the selective bactericidal effect of Chol‐NP to *H. pylori* was confirmed, since the growth of *Escherichia coli* ATCC^®^ 25922™ & *Lactobacillus acidophilus*‐05, bacteria selected as representatives of the gut microbiota, remained unaltered even at 4x the MBC (500 µg mL^−1^), hinting that the dysbiosis side‐effect associated with conventional antibiotherapy is not expected.^[^ ^66^ ^]^

Cholesterol dependence is an attractive therapeutic target to explore in the fight against *H. pylori*. Previous studies on this subject have shown that the extraction of cholesterol from epithelial membranes by *H. pylori* leads to destruction of lipid rafts, modulating the immune system.^[^ ^23^ ^]^ The interaction of *H. pylori* with Chol‐NP, instead of cholesterol from host cells, has the potential of boosting the immune response, while also having bactericidal effect. Still, the interpretation of these results must consider certain limitations, as by being a proof‐of‐concept study, it does not include in vivo experiments. Therefore, the efficacy of Chol‐NP in treating *H. pylori* infection (“Trojan horse” strategy), as well as their safety, biodistribution and bioaccumulation, impact on the gut microbiome and resistance development, will be evaluated in the future and replacing gold nanoparticles by other biomaterials.

## 4 Conclusion

The search for novel antibiotic‐free strategies to address the current anti‐*Helicobacter pylori* conundrum has been on the spotlight. This is a proof‐of‐concept study that demonstrates that cholesterol coated NP are a promising strategy to fight gastric infection. *H. pylori* specifically recognizes and binds to cholesterol exposed from a surface in low concentrations and incorporates Chol‐NP, being killed by membrane disruption. Furthermore, this strategy was effective while having no cytotoxic nor hemolytic effects (at bactericidal and higher concentrations) and showing safety for two bacteria representatives of the gut microbiome. Altogether, these assessments predict that minimal problems and risks will arise in the next stages of testing (animal model).

## 5 Experimental Section

### 2D Self‐Assembled Monolayers (2D Chol‐SAMs) Preparation

Gold substrates (1 × 1 cm^2^ for surface characterization; 0.5 × 0.5 cm^2^ for bacterial assays) were obtained from Instituto de Engenharia de Sistemas e Computadores ‐ Microsistemas e Nanotecnologias, Portugal (INESC‐MN). Before use, gold substrates were cleaned with Piranha solution (7 parts concentrated sulfuric acid (95 vol%, BDH Prolabo), 3 parts hydrogen peroxide (30 vol%, Merck)) for 5 min (caution: this solution reacts violently with many organic materials and should be handled with care). Gold substrates were then sequentially rinsed with absolute ethanol (Merck), Type II water (purified and deionized; resistivity > 1 MΩ cm, conductivity <1 µS cm^−1^ and total organic carbons (TOC) <50 ppb) and absolute ethanol (Merck).

Cholesterol‐poly(ethylene glycol)_75_ thiol/sulfhydryl, MW 3400 Da (Chol‐PEG, Nanocs) and 1‐mercapto‐11‐undecyltetra(ethylene glycol, MW 380.58 (EG4, Sigma‐Aldrich) were prepared as pure solutions at 2 mm in absolute ethanol (Merck). Mixed cholesterol/EG4‐SAMs (Chol‐SAMs) were prepared by immersing the gold coated surfaces in solutions containing different % of Chol‐PEG/EG4 (0 (100% EG4), 12.5, 25, 50, 75 and 100%) with 0.1 mm as final concentration. Incubation was performed at room temperature (RT, ≈25 °C) during 20 h. After incubation, SAMs were rinsed with absolute ethanol, dried with a gentle Argon stream, and stored under inert atmosphere until further use.

### 3D Chol‐SAMs (Cholesterol Nanoparticles (Chol‐NP)) Preparation

Gold nanoparticles (Au‐NP) were prepared as described by Rai et al.,^[^ ^36^ ^]^ resulting in a 1 mg/mL Au‐NP solution. Chol‐NP were obtained by incubating the Au‐NP with 1 mg of Chol‐PEG (24 h at RT) followed by subsequent incubation with 1 mg of EG4 over 4h (Chol‐NP). To attain bare EG4‐NP, 1 mg of EG4 was added to Au‐NP solution and incubated 24 h at RT.

### 5.1 Chol SAMs characterization—Ellipsometry

2D SAMs thickness was determined using an imaging ellipsometer Model EP3 (Nanofilm Surface Analysis) operated in a polarizer‐compensator‐sample‐analyzer (PCSA) mode (null ellipsometry) with a solid‐state laser at 532 nm as light source. The gold substrate refractive index (*n*) and extinction coefficient (*k*) were determined using a delta and psi spectrum with an angle variation between 62° and 76°. Four regions‐of‐interest were used to correct for any instrument misalignment. To determine the thickness of SAMs, the same kind of spectrum was used and *n* and *k* for the organic layer were set as 1.45 and zero, respectively.

Results are the average of 3 measurements with 3 replicates per sample.

### 2D Chol‐SAMs Characterization—Water Contact Angle (WCA)

WCA measurements were done as described^[^ ^30^ ^]^ using the sessile drop method (Data Physics, model OCA 15, equipped with a video CCD‐camera and SCA 20 software). Briefly, 6 µL drops of Type I water (Ultrapure, Milli‐Q, Millipore; resistivity > 18 MΩ cm, conductivity <0.056 µS cm^−1^ and total organic carbons (TOC) <50 ppb) were deposited onto the samples surfaces and images were taken every 2 s over 300 s at RT. Droplet profiles were fitted using the Young‐Laplace formula and the WCA of each sample was calculated by extrapolating the time dependent curve to zero. Results are the average of 2 measurements with 2 replicates per sample.

### 2D Chol‐SAMs characterization—X‐Ray Photoelectron Spectroscopy (XPS)

XPS analysis (Kratos AXIS Ultra HSA) was performed at *Centro de Materiais da Universidade do Porto* (CEMUP). VISION and CasaXPS software were used for data acquisition and analysis, respectively. A monochromatic Al X‐Ray source was used, operating at 15 kV (90 W), fixed analyses transmission (FAT) mode, with a pass energy of 20 eV in the regions of interest, and 80 eV for survey. Data were acquired with a pressure lower than 10^−6^ Pa along a charge neutralization system. Finally, spectra convolution was performed using CasaXPS software for peaks adjustment, via peak fitting with Gaussian‐Lorentzian peak shape and Shirley type background subtraction.

### Chol‐NP Characterization—Dynamic and Electrophoretic Light Scattering Analysis (DLS and ELS)

Chol‐NP as well as controls (Au‐ and EG4‐NP) were diluted 1:50 in Type I Water (Milli‐Q, Millipore), placed in a folded capillary zeta cell and the surface charge (zeta potential, ELS), size and polydispersity index (PdI) (DLS) were measured using a Zetasizer Nano ZS (Malvern Instruments) in triplicate, at an angle of 173° at RT.

NP stability was evaluated in different pH (1.2 and 7.4), following the protocol described by Fonseca *et al*.^[^ ^67^ ^]^ NP surface charge, size and polydispersity index (PdI) were measured before and after incubation at 37 °C for 6 and 24 h, in different solutions: simulated gastric fluid (SGF, pH 1.2, prepared by mixing 0.2 m hydrochloric acid and 0.2 m sodium chloride (both from VWR International)), and phosphate buffered saline (PBS, pH 7.4, prepared by mixing 0.2 m sodium phosphate dibasic dihydrate (Sigma‐Aldrich)) and 0.01 m phosphate buffered saline (Sigma‐Aldrich).

### Chol‐NP Characterization—Transmission Electron Microscopy

Chol‐NP as well as controls (Au‐ and EG4‐NP) were analyzed in terms of morphology with transmission electron microscopy (TEM). Briefly, 5 µL of nanoparticles at 1 mg mL^−1^ were deposited in a TEM grid and allowed to dry. Grids were analyzed in a JEOL JEM 1400 transmission electron microscope (JEOL, Tokyo, Japan) and images were digitally recorded using a CCD digital camera Orius 1100W (Gatan, Tokyo, Japan).

### Helicobacter Pylori Culture

As all *H. pylori* strains are cholesterol dependent, in this proof‐of‐concept study, a highly pathogenic (cagA+ and vacA+) well‐studied and characterized strain was selected.^[^ ^68^ ^]^ *Helicobacter pylori* J99 (ATCC 700824) strain was routinely cultured as described elsewhere.^[^ ^67^ ^]^ Briefly, bacteria were grown in 20 µL spots in Blood Agar (BA; Thermo Scientific) supplemented with 10% (v/v) of defibrinated horse blood (Probiológica) and 0.2% (v/v) antibiotic‐cocktail (polymyxin B, vancomycin, amphotericin B and trimethoprim; all from Sigma‐Aldrich as in^[^ ^69^ ^]^) under microaerophilic conditions (<5% O_2_; GenBox System, BioMérieux) at 37 °C for 48 h. Afterwards, some colonies were streaked onto fresh medium and cultured for another 48 h under the same conditions. Then, bacteria were harvested form the agar plates and the optical density at *λ* = 600 nm (OD_600_; UV/Vis spectrophotometer, Lambda 45, Perkin Elmer) was adjusted to 0.1. Incubation proceeded under microaerophilic conditions, at 150 rpm, 37 °C overnight (≈16 h) in Brucella Broth medium (BB, Oxoid) supplemented with 10% (v/v) of Fetal Bovine Serum (FBS, Gibco).

Prior to each assay, the bacterial inoculum was adjusted to an OD_600_ of 0.03 (≈1 × 10^7^ colony forming units (CFUs) mL^−1^).^[^ ^35^, ^70^ ^]^ The initial inoculum was confirmed by inoculation of 20 µL drops of the bacterial suspension onto BA and, after 5 d of incubation, the CFUs mL^−1^ were determined.

### Escherichia coli and Lactobacillus acidophilus Culture

*Escherichia coli* ATCC^®^ 25922™ strain was streaked and incubated overnight (≈16 h) at 37 °C in Trypticase Soy Agar (TSA, Sigma‐Aldrich). After, a colony was harvested and incubated in Tryptic Soy Broth (Merck), overnight, at 150 rpm, 37 °C.

*Lactobacillus acidophilus*‐05 strain (LA‐5, provided by Chr. Hansen, Hørsholm Denmark) was streaked in De Man, Rogosa and Sharpe agar (MRS agar, Biokar Diagnostics) for 48 h in a microaerophilic environment. Then, a colony was harvested and incubated overnight at 37 °C, 150 rpm in MRS broth (Biokar Diagnostics) in a microaerophilic environment. *E. coli* and *L. acidophilus* inoculums were adjusted to a final concentration of 1×10^5^ CFU mL^−1^ in accordance with the Clinical Laboratory Standards Institute (CLSI) guidelines,^[^ ^49^ ^]^ confirmed by inoculation of 10 µL drops onto TSA and MRS plates, respectively.

### Helicobacter Pylori Recognition of Immobilized Cholesterol

Chol‐SAMs with different % of Chol‐/EG4‐thiols were prehydrated with PBS in a 24‐well plate for 15 min as in Parreira et al.^[^ ^30^ ^]^ Afterwards, PBS was gently removed, and either *H. pylori* (1 × 10^7^ CFU mL^−1^), *E. coli* or *L. acidophilus* (1 × 10^5^ CFU mL^−1^) were incubated with SAMs at 37 °C, 150 rpm for 2 h according to.^[^ ^30^, ^35^ ^]^

### Adherent Bacteria

After incubation, Chol‐SAMs were rinsed thrice with PBS in 5 min intervals per washing, with agitation (150 rpm) at 37 °C. Adhered bacteria were stained with Vectashield with DAPI (Vector Laboratories, Inc.) and images were obtained with an Inverted Fluorescence Microscope (IFM, Zeiss Axiovert 200 MOT) at 400x magnification. Bacterial counts were manually done from the photographs using the ImageJ software in 9 random fields per sample and expressed as number of bacteria mm^−2^. Results were obtained from three independent assays with triplicates.

For the live/dead assay, Chol‐SAMs were rinsed with 0.9% NaCl for 15 minutes to remove non‐adherent bacteria. Adhered *H. pylori* were then stained with LIVE/DEAD BacLight kit (Thermo Fisher Scientific) and mounted with Vectashield (Vector Laboratories, Inc.). Bacteria were visualized with an IFM at 400x magnification. Bacterial counts were manually done from the photographs using the ImageJ software in 9 random fields per sample and expressed as number of bacteria mm^−2^. Results were obtained from 3 independent assays with triplicates.

### Supernatant Bacteria

The bacterial supernatant was removed, serially diluted in PBS and plated on the respective media (BA, TSA or MRS agar plates), and the CFU mL^−1^ determined after 5 d of incubation (*H. pylori*), 24 h (*E. coli*) or 48 h (*L. acidophilus*). Results were obtained from three independent assays with triplicates.

### Cholesterol Competitive Adhesion Assays

The effect of cholesterol depletion from the culture media onto *H. pylori* adhesion to Chol‐SAMs was tested using a *H. pylori* inoculum prepared in Brucella Broth with 1% FBS (instead of 10% FBS; FBS is the medium cholesterol source) and using 0% Chol‐SAMs (EG4‐SAMs), 25% Chol‐SAMs and 100% Chol‐SAMs.

### Chol‐NP Antimicrobial Performance

*H. pylori* was incubated 6 h and 24 h (BB+10%FBS) with different concentrations of Chol‐NP (62.5, 125, 250, 500 and 750 µg mL^−1^) under microaerophilic conditions at 37 °C, 150 rpm. After incubation, samples were serially diluted in PBS and inoculated in BA plates. CFU mL^−1^ were determined after 5 d of incubation at 37 °C under microaerophilic settings.

To assess the specificity of the Chol‐NP, *E. coli* and *L. acidophilus* were incubated with 125, 250, and 500 µg mL^−1^ of Chol‐NP for 2 h, at 37 °C, 150 rpm. Afterwards, samples were serially diluted in PBS, and drop‐plated in TSA for *E. coli*, or MRS agar *for L. acidophilus*. These were incubated for 24 and 48 h, respectively, at 37 °C, after which CFUs were counted and CFU mL^−1^ were determined. Au‐ and EG4‐NP were used as controls and results express three independent assays with triplicates per sample.

### Chol‐NP Mechanism of Action

The interaction of Chol‐NP with the membrane of *H. pylori* was studied with transmission electron microscopy (TEM) following the protocol described.^[^ ^71^ ^]^ Briefly, *H. pylori* was incubated with Chol‐NP (as well as with the controls) at 125 µg mL^−1^ for 2, 6, and 24 h, at 37 °C in microaerophilic conditions. To ensure enough bacterial content for posterior sample preparation, 10 replicates of each condition were prepared. Then, all the replicates per each condition were combined and centrifuged (3000× *g*, RT, 10 min). The bacterial pellets were fixed with a solution of 2% glutaraldehyde, 2.5% formaldehyde (both from Electron Microscopy Sciences) and 0.1 m sodium cacodylate buffer (pH 7.4) for 1 h at RT. Samples were post‐fixed with 1% osmium tetroxide (Electron Microscopy Sciences) diluted in 0.1 m sodium cacodylate buffer and re‐suspended in Histogel (HG‐4000‐012, Thermo Fisher Scientific). Afterward, they were stained with aqueous 1% uranyl acetate solution overnight, dehydrated with ethanol and propylene oxide and then embedded in Embed‐ 812 resin (Electron Microscopy Sciences). Each resin was cut in ultrathin sections of 50 nm thickness on an RMC Ultramicrotome (PowerTome, Moses Lake, WA, USA) using Diatome diamond knives (Delaware Diamond Knives, Wilmington, DE, USA) and mounted on mesh nickel grids (Electron Microscopy Sciences). Next, the sections were stained with uranyl acetate substitute and lead citrate (both from Electron Microscopy Sciences) for 5 min each. Ultrathin sections were analyzed in a JEOL JEM 1400 transmission electron microscope (JEOL, Tokyo, Japan) and images were digitally recorded using a CCD digital camera Orius 1100W (Gatan, Tokyo, Japan).

### Cytotoxicity Assay

AGS cells (ATCC^®^ CRL‐1739™) were grown in Roswell Park Memorial Institute 1640 medium (RPMI, Corning) supplemented with L‐glutamine, 10% (v/v) FBS and 1% (v/v) Penicillin‐Streptomycin (Pen/Strep, Biowest) (Full RPMI) at 37 °C in a 5% CO_2_ atmosphere, until 70–80% confluence was achieved.^[^ ^72^ ^]^

Cells at 70–80% confluence were detached with trypsin (Biowest) (37 °C, 5% CO_2_ for 5 min) and seeded at 1×10^4^ cells in 96‐well plates (SARSTEDT, TC Plate 96 Well, Suspension, F) over 24 h, 37 C, 5% CO_2_ atmosphere.

Chol‐NP (125, 250, 500 and 750 µg/mL) and controls (Au‐ and EG4‐NP) cytotoxicity was evaluated in accordance with the ISO 10993–5:2009 standard.^[^ ^50^ ^]^ Briefly, 300 µL of each NP was centrifuged (10 min 14 000 rpm; Spectrafuge 16M, National Labnet Co.) and resuspended in Full RPMI media. Cell metabolic activity was assessed via resazurin assay using a microplate fluorometer (Synergy Mx; BioTek, *λ*_Ex_ = 530 nm, *λ*_Em_ = 590 nm).

Cell metabolic activity was calculated using Equation 1:

(1) $$\%\;\mathrm{Cell}\;\mathrm{metabolic}\;\mathrm{activity}=\frac{F_{\mathrm{ex}}-\;F_{\mathrm{blank}}}{F_{\mathrm{control}}-\;F_{\mathrm{blank}}}\;\times 100$$

with *F*_exp_ being the fluorescence of AGs cells exposed to the NP, *F*_blank_ the fluorescence of the Full RPMI medium, and *F*_control_ AGS cells (positive control). Results express three independent assays with triplicates.

### Hemocompatibility

Chol‐NP hemocompatibility was determined through a hemolysis assay adapted from.^[^ ^73^ ^]^ Briefly, red blood cells (RBCs) were isolated from buffy coats (obtained from the Immunohemotherapy Service, Hospital S. João, Porto, Portugal) by centrifugation (400 *g*, RT, 30 min) over a density gradient with Histopaque‐1077 (Sigma–Aldrich), removal of the plasma upper layer and three washes with PBS (370*g*, RT, 10 min).

RBCs concentration was adjusted to 2 × 10^8^ cells mL^−1^ and these were incubated with Chol‐NP (125 and 250 µg mL^−1^), Au‐and EG4‐ NP (125, 250, and 500 µg mL^−1^) to include the respective MBC, for 3 h at 37 °C in round bottom 96 wells polypropylene microtiter plates (Corning). After, plates were centrifuged (2250 *g*, RT, 15 min) and the supernatant was collected to black polypropylene 96 wells microtiter plates (Corning) for absorbance reading at 380 (A380), 415 (A415), and 450 nm (A450) using a micro‐plate reader spectrophotometer (Synergy Mx; BioTek).

The amount of hemoglobin (Hb) was calculated using Equation 2:

(2) $$\begin{array}{c} \mathrm{Hb}\;\mathrm{value}\;\mathrm{of}\;\mathrm{sample}\;\;\left(\frac{\mathrm{mg}}{\mathrm{dL}}\right)=\left[\left[2\times A415-\left(A380+A450\right)\right]\right.\\ \left.\times 1000\times \mathrm{dilutionfactor}\right]\;/E \end{array}$$

A415 is the Soret band absorption of Hb and A380, A450 are correction factors of uroporphyrin, whose absorption falls under the same wavelength range. *E* is the molar absorptivity of oxyhemoglobin at 415 nm, which is 79.46. The hemolytic potential of the NP was calculated using Equation 3:

(3) $$\mathrm{Hemolysis}\;\;\left(\%\right)=\frac{\mathrm{Hb}\;\mathrm{value}\;\mathrm{of}\;\mathrm{sample}}{\mathrm{Total}\;\mathrm{Hb}\;\mathrm{value}}\;\times 100$$

Total Hb value corresponds to 100% hemolysis with Triton 1% (Sigma Aldrich, X100). Assays were performed in triplicate and repeated three times.

### Statistical Analysis

Statistical analysis was performed using the One‐way ANOVA, followed by Dunnett's or Tukey's multiple comparisons test, or unpaired T‐test and unpaired T‐test with Welch's correction using GraphPad Prism 8 (version 8.0.1). Statistically significant differences were considered for *p*<0.05.

## Conflict of Interest

The authors declare no conflict of interest.

## Supporting information



Supporting Information

## Data Availability

The data that support the findings of this study are available from the corresponding author upon reasonable request.
