# Supplementary material for: Cholesterol Functionalized Nanoparticles Are Effective against Helicobacter pylori, the Gastric Bug: A Proof‐of‐Concept Study
Source: Adv Healthc Mater. 2025 Feb 5;14(10):2404065. doi: 10.1002/adhm.202404065 (PMC12004443; doi:10.1002/adhm.202404065)
Supplement: Supplementary file 1 — Supporting Information [file ADHM-14-0-s001.docx]

Supporting Information

Cholesterol functionalized nanoparticles are effective against *Helicobacter pylori*, the gastric bug: a proof-of-concept study

Ana Sofia Pinho, Renato Pereira, Mariana Pereira, Akhilesh Rai, Lino Ferreira, Maria Cristina Martins and Paula Parreira *


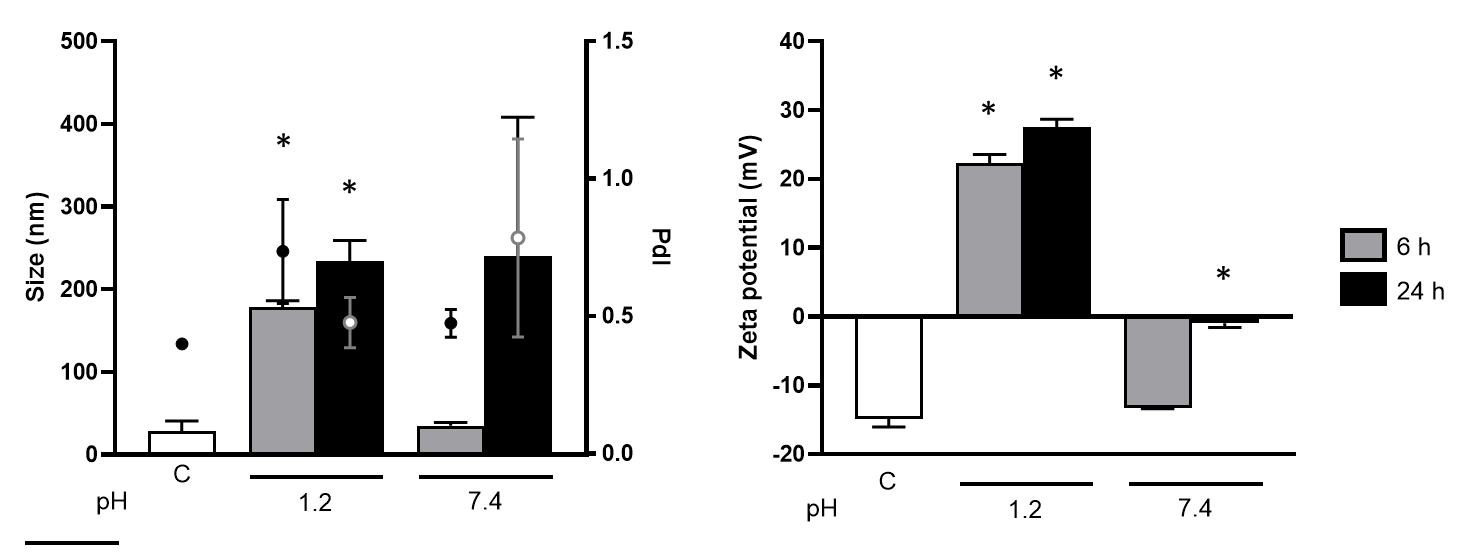


**Figure S1**. EG4-NP characterization at different pH. pH 1.2 (simulated gastric fluid, SGF) and 7.4 (phosphate buffer saline, PBS). C – controls measured before incubation at different pH. Statistical analysis was performed using the unpaired T-test. n=3. *Statistically significant different from the control (C) (p<0.05).

**Figure S2**. Adhesion of bacteria representative of the gut microbiota to Chol-SAMs after 2 h. *E. coli* (**a**) adhered to Chol-SAMs and (**b**) planktonic cells; *L. acidophilus* (**c)** adhered to Chol-SAMs, (**d**) planktonic cells. * Statistically significant different from the control surface 0% Chol-SAMs (EG4-SAMs); ($p$<0.05).

**Figure S3.** (**a**) *E. coli a*nd (**b**) *L. acidophilus* CFU/mL after 2 h of incubation with Chol-, EG4- and Au-NP. Incubation time was adjusted to 2h due to the bacteria doubling time of circa 20 minutes in optimal conditions versus the 6h doubling time of *H. pylori*.

Using a different inoculum concentration (1 x 10^7^ CFU/mL instead of 1 x 10^5^ CFU/mL used to comply with the CLSI guidelines) did not impact the outcome, as the order of magnitude of adhered bacteria to the surfaces remained the same (data not shown).
